# Oncotargeting G proteins: The Hippo in the room

**DOI:** 10.18632/oncotarget.2815

**Published:** 2014-11-25

**Authors:** Xiaodong Feng, Qianming Chen, J. Silvio Gutkind

**Affiliations:** ^1^ Oral and Pharyngeal Cancer Branch, National Institute of Dental and Craniofacial Research, National Institutes of Health, Bethesda, MD, USA; ^2^ State Key Laboratory of Oral Diseases, West China Hospital of Stomatology, Sichuan University, Chengdu, Sichuan, China

**Keywords:** YAP, GNAQ, GNA11, Melanoma, Cancer, Signal transduction, Rho GTPases

The core components of the Hippo pathway are conserved from flies to mammals [1]. In humans, these include a kinase cascade initiated by the Hippo kinase MST1/2 associated with the adaptor protein WW45/SAV1, and LATS1/2 in complex with MOB1, which in turn, phosphorylates and inhibits the mammalian transcription co-activator YAP and its related protein TAZ [1]. YAP plays a critical role in organ size control during development, and its persistent nuclear localization and activation contributes to multiple human malignancies [1]. The mechanisms driving YAP activation in most cancers, however, are often not clearly understood. In recent studies [2, 3], we and Guan's team found that YAP activation represents a key molecular event contributing to uveal melanoma, the most frequent ocular malignancy in adults. Uveal melanoma growth is driven by gain-of-function mutations in *GNAQ* or *GNA11* oncogenes, encoding persistently active G protein α subunits of the Gq family [4]. As the signaling capacity of G proteins and their coupled receptors (GPCRs) has been extensively investigated, these findings provided an opportunity to identify cancer-associated mechanisms resulting in YAP activation, and to explore whether YAP represents a suitable oncotarget for cancer treatment.

**Figure 1:**
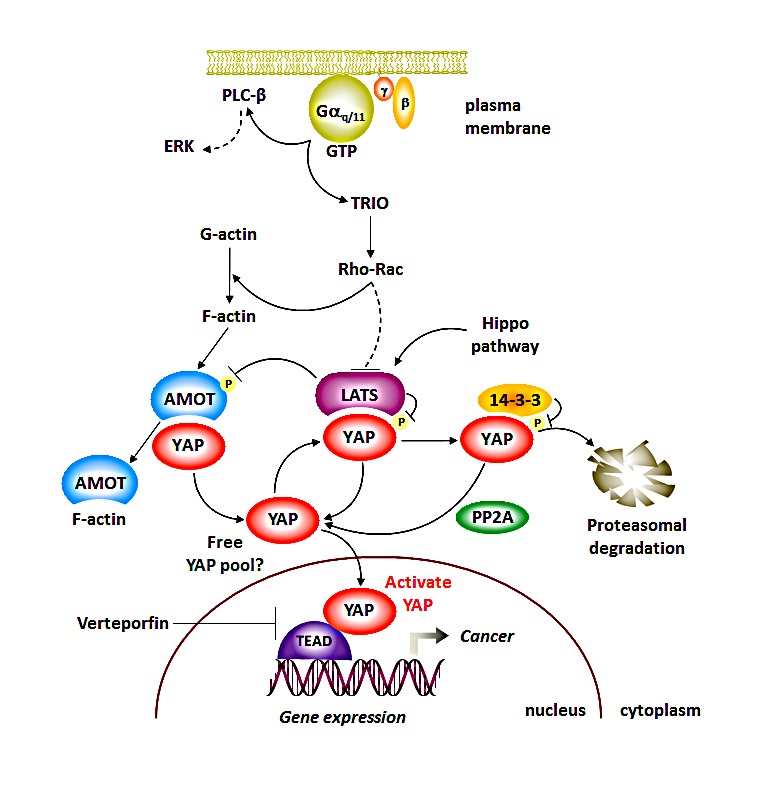
The *GNAQ* and *GNA11* uveal melanoma oncogenes encode persistently activated heterotrimeric G protein α subunits of the Gαq family Gαq activates classical cytosolic second messengers as well as guanine nucleotide exchange factors activating the small GTPases Rho and Rac, which in turn promote the stimulation of the YAP transcriptional co-activator by a cytoskeletal-mediated mechanism resulting in YAP dissociation from AMOT and its related proteins. The dynamic regulation of YAP molecular complexes and its distinct pools by the canonical Hippo pathway and Gαq, and the therapeutic potential of targeting active nuclear YAP are described in the text.

The oncogenic potential of *GNAQ* was initially revealed by a systematic analysis of the transforming potential of G proteins and GPCRs [5]. Gαq stimulates PLCβ and the consequent increase in cytosolic Ca^2+^ levels and diacylglycerol (DAG) production, which stimulate classical PKCs and ERK (Fig. 1). The latter mimics the impact of *B-RAF* or *N-RAS* oncogene mutations in cutaneous melanomas [4]. However, inhibitors of the ERK pathway increase progression free survival but has a limited impact in overall survival of uveal melanoma patients [6], suggesting that *GNAQ* can activate oncogenic signaling circuitries circumventing ERK inhibition. In this regard, a genome wide screen revealed that the activation of growth promoting gene programs by Gαq involves the stimulation of Rho GTPases through the direct activation of a guanine nucleotide exchange factor known as Trio [5]. Indeed, we found that YAP activation by *GNAQ* is dependent Trio and its regulated Rho GTPases, RhoA and Rac1, but not on PLC-generated second-messengers [2].

The detailed analysis of YAP activation by *GNAQ* in uveal melanoma helped identify a novel signaling mechanism controlling YAP function. Specifically, while Gq-coupled GPCRs diminish the negative phosphorylation of YAP by inhibiting LATS, in uveal melanoma cells LATS1/2 remains partially active, hence YAP dephosphorylation may not be sufficient to explain its overactivity [2]. We found that the accumulation of polymerized F-actin upon Rho-GTPase activation is critical for YAP stimulation by *GNAQ* [2], aligned with the role of F-actin in YAP activation during mechanosensing signaling (reviewed in [7]). In search for the underlying mechanism, we found that F-actin accumulation causes the release of YAP bound to AMOT, thereby promoting an increase in the free-YAP pool that can then translocate to the nucleus and regulate gene expression [2].

Our study [2] and recent reports (reviewed in [7]) provided a new mechanistic insight into how cytoskeletal changes can regulate YAP function. YAP (and TAZ), are part of multiple cytosolic protein complexes established by the direct interaction between the WW domains of YAP with PPxY motifs found in most YAP-associated proteins, including LATS and AMOT (reviewed in [7]). YAP binding to LATS facilitates YAP phosphorylation and its subsequent inactivation by the association of phospho-YAP with 14-3-3 or its degradation by the proteasome. Instead, AMOT inhibits nuclear YAP function by sequestering it in the cytosol (reviewed in [7]). As AMOT's PPxY motifs are adjacent to its F-actin binding region, polymerized actin competes for YAP binding thereby increasing free YAP, while actin depolymerization and increase in G-actin will result in the accumulation of inactive, AMOT-bound YAP protein complexes [2, 7] (Fig. 1).

These YAP pools are likely dynamically regulated (Fig. 1). AMOT represses YAP but competes for LATS binding to YAP, hence protecting YAP from its inactivation by LATS. LATS can also phosphorylate AMOT, preventing its binding to F-actin (reviewed in [7]), thus providing a feedback mechanism favoring the stability of the AMOT-YAP transcriptionally inactive pool. Robust activation of cytoskeletal changes can however result in the dissociation of YAP from AMOT (and its related AMOTL1 and AMOTL2), suggesting that AMOT may act as a YAP inhibitor or facilitate YAP activation depending on the status of actin polymerization. In turn, the interplay between these distinct cytosolic and nuclear YAP pools may help explain how actin polymerization controls YAP during the transduction of mechanosensing signals. As AMOT orthologs are not found in Drosophila, additional mechanisms might exist controlling the interplay between YAP pools in flies and perhaps in mammals, which warrants further investigation.

These findings may have direct clinical relevance, as recent drug screens revealed that a family of porphyrin-related molecules can inhibit the interaction of YAP with TEAD transcription family members [8]. Among them, verteporfin (VP) is already a FDA-approved drug for eye disease indications such as macular degeneration. Remarkably, VP can potently inhibit uveal melanoma tumor growth in experimental systems [2, 3], suggesting that YAP may represent a suitable therapeutic target for the treatment of uveal melanoma and other human malignancies characterized by unrestrained YAP function.
